# Why African Americans Are a Potential Target for COVID-19 Infection in the United States

**DOI:** 10.2196/19934

**Published:** 2020-06-12

**Authors:** Alireza Hamidian Jahromi, Anahid Hamidianjahromi

**Affiliations:** 1 Department of Plastic Surgery The University of Tennessee Health Science Center Memphis, TN United States; 2 Jahrom University of Medical Sciences Jahrom Iran

**Keywords:** coronavirus, COVID-19, African American, mortality, race, virus, minority, infectious disease

## Abstract

Since the World Health Organization declared the coronavirus disease (COVID-19) outbreak a pandemic, significant changes have occurred in the United States as the infection spread reached and passed its exponential phase. A stringent analysis of COVID-19 epidemiologic data requires time and would generally be expected to happen with significant delay after the exponential phase of the disease is over and when the focus of the health care system is diverted away from crisis management. Although much has been said about high-risk groups and the vulnerability of the elderly and patients with underlying comorbidities, the impact of race on the susceptibility of ethnic minorities living in indigent communities has not been discussed in detail worldwide and specifically in the United States. There are currently some data on disparities between African American and Caucasian populations for COVID-19 infection and mortality. While health care authorities are reorganizing resources and infrastructure to provide care for symptomatic COVID-19 patients, they should not shy away from protecting the general public as a whole and specifically the most vulnerable members of society, such as the elderly, ethnic minorities, and people with underlying comorbidities.

Although it has only been a short time since March 11th, 2020, when the World Health Organization officially announced that the coronavirus disease (COVID-19) outbreak had reached the level of a pandemic [1], things have changed significantly for the United States. The country has reached and passed the exponential phase of the COVID-19 pandemic, with over 1.7 million confirmed cases and close to 105,000 COVID-19–related deaths [2]. These numbers seem to reflect only the tip of the iceberg; the figures would be much higher if we are to include asymptomatic carriers and mildly symptomatic untested cases in the population. As shown in a study by Ing et al [3], testing all of the passengers of a cruise ship revealed a ratio of asymptomatic COVID-19–positive patients to symptomatic patients to be 4:1. Furthermore, a second wave of COVID-19 infection is always a possibility.

A stringent analysis of COVID-19 epidemiologic data requires time and would generally be expected to happen with some delay after the exponential phase of the disease is over and when the focus of the health care system is not on crisis management. Although much is said about high-risk groups and the vulnerability of the elderly and patients with underlying comorbidities (eg, hypertension, diabetes, malignancies, cerebrovascular disease, and underlying pulmonary/cardiovascular disease like chronic obstructive pulmonary disease and asthma [4-6]), the impact of race on the susceptibility of ethnic minorities living in indigent communities has not been discussed in detail worldwide and in the United States. Myers [7] and Dyer [8] brought up this issue and discussed as to why, in their opinion, ethnic minorities, in particular African American or black populations, are more vulnerable to COVID-19 infection and mortality. Dyer [8] also brought up the same issue with other “invisible communities” (ie, Navajo Nations communities of Arizona, New Mexico, and Utah and “undocumented immigrants”) and proposed delayed health care seeking behavior, inferior care, difficulty in trusting authorities (eg, fear of charges against those without legal immigration documents upon receiving care or being tested for COVID-19) as possible explanations. Dyer [8] believes that African Americans are generally suspicious of the health care system following the Tuskegee syphilis scandal (1932-1972), where black participants with a syphilis diagnosis were monitored for almost 40 years and left untreated despite the availability of treatment for syphilis at the time; this mistrust continues to impact medical advice–seeking behaviors among black populations in the United States [8].

Knowledge of comorbid risk factors as well as vulnerable groups can be useful for clinicians in directing early and appropriate medical management and allocating resources when handling patients with COVID-19. As “ethnicity” is still considered a sensitive/controversial topic in the United States, its inclusion as a research topic as well as patients’ demographic data should be cautiously evaluated; even the Centers for Disease Control and Prevention does not include race and ethnicity information in their publicly available data when presenting the demographic and geographic distributions of COVID-19 cases. Myers [7] provided invaluable, practical recommendations to health care authorities and hospital administrations caring for vulnerable populations with COVID-19, including patients with comorbidities and ethnic minorities. Cooney [9] presented examples from Illinois, Michigan, and Milwaukee County in Wisconsin to show disparities and the overrepresentation of COVID-19 cases and mortalities among African Americans compared to other ethnic groups, especially Caucasians. Although these areas do not constitute the majority of the United States, these are a handful of exceptional areas where the local government agencies have included race as part of the shared information when presenting the most vulnerable and affected segments of society “hardest hit by the virus” [9,10]. While African Americans in Illinois, as an example, account for 17% of confirmed COVID-19 cases and 29.7% of attributed deaths, they make up only 15% of the state’s population [10]. In Louisiana, based on the 2019 US census, 33% of the population is African American, but this group accounts for 55% of COVID-19–related deaths, according to data from the Department of Health [11]. North Carolina, Michigan, St Louis, New York City, and Alabama are other examples of disparities in COVID-19–related deaths and ethnicity, with higher death rates reported in African Americans compared to Caucasians [12-15].

This disparity is not a confirmed fact across the board, but it is present in some states as mentioned above. There are some possible explanations for this pattern. The proposed association between COVID-19 and angiotensin-converting enzyme II (ACE-II) secondary to ACE-II being a co-receptor for respiratory viral entry, along with potential innate ACE-II receptor differences in function, response to medications, and gene polymorphism in the African American population compared to other ethnic groups [16,17] could be an explanation that needs further investigation. Having higher incidence of hypertension, diabetes, malignancies, heart failure, cerebrovascular disease, and underlying pulmonary/cardiovascular disease could also make the African American community a potential high-risk group for COVID-19 [7,8,18,19]. Higher BMI among African Americans compared to other groups, particularly Caucasians, has been shown to be present in children and adults [20,21]. This contributes to a general decrease in self-rated health among older African Americans and can even interfere with the protective effects of education and income [22]. Higher average BMI in African Americans could potentially decrease their survival rate in cases with COVID-19–related acute respiratory distress syndrome and increase the rate of associated complications [23]. Other possible contributing factors to COVID-19 susceptibility among African Americans include lower socioeconomic status, crowded living conditions, living in densely populated parts of cities, reduced access to hygienic products and personal protection equipment, unemployment, employment in the more virus-exposed fields of the job market, along with cultural habits that hinder medical advice seeking (although their exact role warrants further investigations) [24]. Myers [7] went a step further and proposed the influence of “systemic racism on baseline health” as a compounding factor influencing the health of ethnic minorities including African American people. While health care authorities are reorganizing resources and infrastructure to provide care for symptomatic COVID-19 patients [25], they should not forgo protecting the general public as a whole and specifically the most vulnerable members of society, such as the elderly, ethnic minorities, and people with underlying comorbidities. We agree with Myers [7] and Fouad et al [26] that the following are essential steps toward a fairer and more effective health care system when battling COVID-19 infection: collecting data on patient care during the COVID-19 era including race, implementation of social policies to reduce economic burden, culturally sensitive and appropriate messaging tailored to certain communities, increased accessibility of testing and vaccination (after vaccine development) for vulnerable communities, real-time reporting of disparities and reflective redistribution of resources, aggressive treatment of comorbidities simultaneously while treating COVID-19 infections, and prioritization of community-based health care systems that primarily serve ethnic minorities. We can turn this crisis into an opportunity to reduce disparities in care in our health care system [26].

## Abbreviations

ACE-II: angiotensin-converting enzyme II
COVID-19: coronavirus disease
